# Physiological Response of Spotted Seabass (*Lateolabrax maculatus*) to Different Dietary Available Phosphorus Levels and Water Temperature: Changes in Growth, Lipid Metabolism, Antioxidant Status and Intestinal Microbiota

**DOI:** 10.3390/antiox12122128

**Published:** 2023-12-16

**Authors:** Jiarong Guo, Ling Wang, Kai Song, Kangle Lu, Xueshan Li, Chunxiao Zhang

**Affiliations:** 1 State Key Laboratory of Mariculture Breeding, Fisheries College, Jimei University, Xiamen 361021, China; guojr9913@163.com (J.G.); lukangle@jmu.edu.cn (K.L.); 2 Xiamen Key Laboratory for Feed Quality Testing and Safety Evaluation, Fisheries College, Jimei University, Xiamen 361021, China; 3 Key Laboratory of Healthy Mariculture for the East China Sea, Ministry of Agriculture and Rural Affairs, Jimei University, Xiamen 361021, China

**Keywords:** *Lateolabrax maculatus*, phosphorus, water temperatures, physiological metabolism, antioxidant status

## Abstract

A 10-week growth experiment was conducted to assess the physiological response of spotted seabass (*Lateolabrax maculatus*) raised at moderate (27 °C) and high temperatures (33 °C) to different dietary available phosphorus (P) levels. Five diets with available P levels of 0.35, 0.55, 0.71, 0.82 and 0.92% were formulated, respectively. A water temperature of 33 °C significantly decreased growth performance and feed utilization, and increased oxidative stress and lipid deposition of spotted seabass compared with 27 °C. A second-order polynomial regression analysis based on weight gain (WG) showed that the available P requirement of spotted seabass raised at 27 °C and 33 °C was 0.72% and 0.78%, respectively. The addition of 0.71–0.82% P to the diet improved the growth performance, feed utilization, and antioxidant capacity of spotted seabass and alleviated the excessive lipid deposition compared with the low-P diet (0.35% P). Moreover, the addition of 0.71–0.92% P to diets increased the diversity of intestinal microbiota and the relative abundance of *Lactococcus lactis* and decreased the relative abundance of *Plesiomonas* compared with the low-P diet. Thus, dietary supplementation with 0.71–0.82% P improved the growth performance, antioxidant capacity and microbial composition of spotted seabass, and alleviated the disturbance of lipid metabolism caused by high temperature or low-P diet.

## 1. Introduction

Phosphorus (P) is an essential nutrient for animals because it is required for bone tissue formation and maintenance, phospholipid and nucleic acid biosynthesis, intracellular signaling, and other metabolic processes [1,2,3,4]. As fish can only absorb minor amounts of P from the water, diet is the primary source of P for fish to meet their nutritional needs [5]. P levels in the diet not only directly affect fish health but are also economically and environmentally critical [6]. On the one hand, P deficiency decreased fish appetite, growth, feed efficiency and antioxidant capacity, but increased lipid deposition and improper bone mineralization [7,8]. On the other hand, when aquatic animals consume excess P, some of it will be discharged into the water, leading to eutrophication [9]. With the promotion of precision nutrition, accurately determining the P requirements of fish can maximize the conservation of resources and protect the environment while ensuring fish health and growth.

Water temperature, as an important environmental factor, has a direct impact on fish growth, antioxidant capacity and immunity [10,11]. Fish’s growth, development, feeding, and reproduction are put to the test when they are exposed to uncomfortably warm water [12]. In addition, the nutrient requirements of fish vary under different water temperature conditions [13]. Wang et al. [14] reported that spotted seabass increased dietary iron requirements to meet iron metabolic homeostasis under high-temperature conditions. The nutritional status of fish following changes in ambient temperature is expected to be regulated by physiological metabolic processes of energy and other nutrients in the fish body [15]. However, the potential impacts of temperature on fish dietary P requirements and P turnover metabolism have long been disregarded.

Fish’s intestine is an organ responsible for digestion and absorption of nutrients, and it also serves as the primary site for colonization and life of microbial communities [16]. Changes in diet and environment have a significant impact on the community structure of their intestinal microbiota [17,18]. It has been reported that appropriate P levels can promote the development of fish intestinal microbiota, increase the number of beneficial bacteria, regulate the intestinal microecological balance, and maintain intestinal health [19,20]. In addition, temperature changes can also affect the composition of intestinal microbiota [21]. Zhao et al. [22] observed significant differences between the fecal microbiota of fish exposed to different water temperatures. However, the combined effects of P and water temperature on fish intestinal microbiota are still poorly understood.

Spotted seabass (*Lateolabrax maculatus*) is a carnivorous fish that is widely reared in southern China. It is a temperate fish with an optimum water temperature range of 16–28 °C [23]. However, summer water temperatures in southern China often exceed 30 °C, causing significant economic losses to the spotted seabass culture industry. Studies on the relationships between dietary P and water temperature for this species are not currently available. Therefore, the aim of this study was to investigate the available P requirements of spotted seabass raised at moderate (27 °C) and high (33 °C) water temperatures, and to assess the effects of different available P levels and water temperatures on the growth, lipid metabolism, antioxidant capacity, and intestinal microbiota of spotted seabass.

## 2. Materials and Methods

### 2.1. Diets and Feeding Experiment

Five experimental diets were prepared by adding sodium dihydrogen phosphate and dipotassium hydrogen phosphate (NaH_2_PO_4_ and K_2_HPO_4_, AR, Sinopharm Chemical Reagent Co., Ltd., Beijing, China) to the basal diets, and the available P contents of the prepared diets were 0.35, 0.55, 0.71, 0.82 and 0.92%, respectively. The formulation and approximate makeup of diets are provided in Table S1, and the preparation of the diet was based on previous research carried out in our laboratory [24].

This study was carried out according to the guidelines of the Declaration of Helsinki and was approved by the Animal Care Advisory Committee of Jimei University (Approval No. 2019-0906-003, 6 September 2019). The feeding experiment was conducted in Jimei University’s recirculating aquaculture system. Spotted seabass juveniles were provided by a fish hatchery (Zhangzhou, China). After the acclimatization phase, 900 healthy, uniformly proportioned fish with a mean body weight of 3.53 ± 0.34 g were randomly assigned to 30 identical 200 L fiberglass fish tanks. The voltage water heater (dH-1000, D&A, Essen, German) raised the temperature of the aerated tap water to either 27 °C or 33 °C before the experiment at a rate of 1 °C each day. The experiment lasted for 10 weeks, and the fish were hand-fed twice daily (8:30 and 17:30) to ensure apparent satiation. Approximately 40% of the total storage water was replaced each day, and the water to be replaced was heated to the required temperature in advance. The following water characteristics were kept constant during the feeding test: pH 6.8–7.2 (S8-meter, Mettler-toledo, Columbus, USA), and dissolved oxygen > 6.5 mg/L (DO-200, Harveson, Suzhou, China).

### 2.2. Sample Collection

The fish were starved for 24 h before being weighed to determine the growth parameters. Before being sampled for examination, the selected fish were euthanized with 100 mg/L MS-222 (Sigma, Ronkonkoma, NY, USA). Three fish were randomly selected from each tank and kept in a −20 °C refrigerator for body composition analysis. Twelve fish per tank were sampled to estimate the morphological parameters by the weight of liver, viscera and intraperitoneal lipid. Using a 27-gauge needle and a 1 mL syringe, blood samples were taken from the caudal veins of 12 fish per tank, and clotted overnight at 4 °C. The serum was collected after centrifugation (3600 r/min, 10 min, 4 °C; 3-30KS, Sigma, USA) and stored at −80 °C until use. Three liver samples per tank were fixed in 10% formaldehyde solution for histological analyses. The intestine and liver samples of the remaining fish were first placed in 2.0 mL EP tubes after collection, then promptly frozen in liquid nitrogen and stored at −80 °C for further examination. In the final week of the trial, the feces in each tank were additionally siphoned for digestibility tests.

### 2.3. Biochemical Parameter Measurements

Commercial kits (Nanjing JianCheng Bioengineering Institute, Nanjing, China) were used to measure total antioxidant capacity (T-AOC), contents of triacylglycerols (TG), P, calcium and malondialdehyde (MDA) as well as activities of alkaline phosphatase (ALP), glutamic pyruvic transaminase (ALT), glutamic oxaloacetic transaminase (AST), catalase (CAT), superoxide dismutase (SOD), glutathione peroxidase (GSH-PX). The experimental procedures were carried out according to the manufacturer’s instructions.

### 2.4. Proximate and Histological Analysis

The moisture content of the samples was determined by drying them at 105 °C until a constant weight was reached. Crude protein was determined using a Dumas nitrogen analyzer (Rapid N III, Frankfurt, Germany), crude lipid was extracted using anhydrous ethanol (Sinopharm Chemical Reagent Co., Ltd., Beijing, China), and ash was determined via incineration in a muffle furnace (550 °C, 8 h, SX2, Yiheng, Shanghai, China). Inductively coupled plasma atomic emission spectrometry (Prodigy Plus, ICP-OES, Leeman, Milwaukee, USA) was used to determine the P content of fish, diet, and manure. The equations for calculating the apparent digestibility coefficient (ADC) of P in experimental diets were as follows [25]:

ADC of P (%) = 100 − 100 × (Y_2_O_3_ in diet/Y_2_O_3_ in feces)% × (P in feces/P in diet)%.

Available P = Total P in diet × ADC of P in diet.

The oil red O staining was conducted as described in a previous study [26].

### 2.5. Real-Time Quantitative PCR

Total RNA from the liver and intestine was extracted and subjected to RT-PCR following the procedures described in our previous study [27]. Primers were created based on the transcriptome of spotted seabass (Table S2). The relative expression levels of genes were determined using the 2^−ΔΔCt^ method [28].

### 2.6. Analysis of Intestinal Microbiota

Using the HiPure Soil DNA Kit (Magen, Guangzhou, China), bacterial DNA was extracted and sent for sequencing of the V3–V4 regions of the 16S rRNA gene on an Illumina MiSeq PE 250 (Gene Denovo Biotechnology Co., Ltd., Guangzhou, China).

### 2.7. Statistical Analysis

Using the SPSS software version 22.0, one- and two-way ANOVA analyses were performed on the data to determine whether there were significant differences due to the available P level, water temperature or the interaction (IBM, New York, NY, USA). Turkey’s test was used to evaluate the difference in means after the ANOVA analysis had revealed the differences between groups. Statistics were considered significant at *p* < 0.05. The second-order polynomial regression model [29] was used to estimate the dietary P requirement for spotted seabass based on weight gain (WG). In addition, Welch’s *t*-test was employed in the Project R Vegan module (version 2.5.3) to compute the alpha diversity index, species comparisons and identify biomarkers with relative abundance above 0.1% between groups.

## 3. Results

### 3.1. Growth Performance and Feed Utilization

Except for FBW and WG, no significant interactions were found between water temperature and available P levels on SR, FR or FCR of spotted seabass. Simple effects analysis showed that FBW and WG showed the highest value (69.78 g and 1883.95%) in spotted seabass fed the diet with 0.71% available P level at 27 °C among the 10 treatments.

Irrespective of available P levels, FBW and WG of spotted seabass raised at 33 °C were significantly lower, while FR and FCR were significantly higher than those of spotted seabass raised at 27 °C (*p* < 0.05). No significant effect was found in SR between spotted seabass raised at 27 °C and 33 °C.

**Figure 1:**
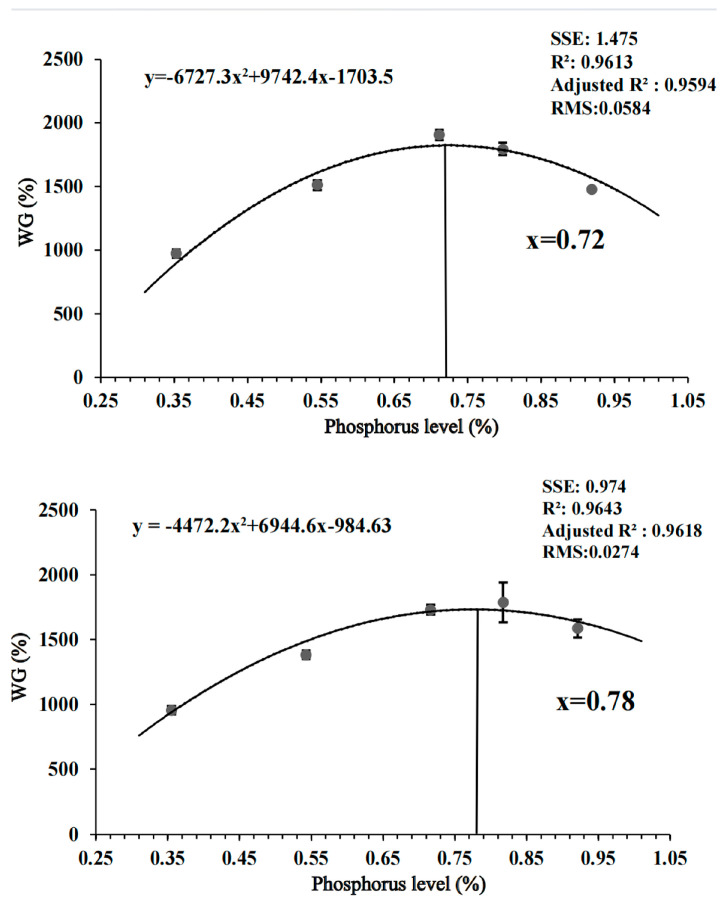
Effects of dietary available phosphorus levels and water temperature on the weight gain (WG) of spotted seabass (*Lateolabrax maculatus*) based on second−order polynomial regression analysis. SSE, the sum of squares dueto error; R^2^, R-square; Adjusted R^2^, Adjusted R-square; RMS, root mean squared.

Irrespective of water temperature, compared to spotted seabass fed diets with 0.35%, 0.55% and 0.92% available P levels, spotted seabass fed diets with 0.71% and 0.82% available P levels had significantly higher FBW and WG and significantly lower FCR (*p* < 0.05). The lowest FBW, WG and SR and the highest FCR were found in spotted seabass fed the diet with 0.35% available P level among dietary treatments (Table 1). A second-order polynomial regression based on WG showed that the available P requirement of spotted seabass was 0.72% and 0.78% at 27 °C and 33 °C, respectively (Figure 1).

### 3.2. Morphological Parameters and Body Compositions

A significant interaction was found between water temperature and available P levels on IFR (*p* < 0.05). Simple effects analysis showed that IFR had the highest value (8.82%) in spotted seabass fed the diet with 0.82% available P level at 33 °C among the 10 treatments. No significant interactions were found between water temperature and available P levels on contents of moisture, ash, lipid or protein in the whole body of spotted seabass (*p* > 0.05).

Irrespective of available P levels, HSI, VSI, CF, IFR and contents of moisture, lipid and ash in the whole body of spotted seabass raised at 33 °C were significantly increased, while the content of whole-body protein was significantly decreased compared with those of spotted seabass raised at 27 °C (*p* < 0.05). Irrespective of water temperature, compared to spotted seabass fed the diet with 0.71% available P level, spotted seabass fed the diet with 0.35% available P level had significantly higher IFR, whole-body moisture and lipid contents, and significantly lower whole-body ash and protein contents (*p* < 0.05). In addition, compared to spotted seabass fed diets with 0.35% and 0.55% available P levels, spotted seabass fed the diet with 0.92% available P level had significantly higher whole-body ash content and significantly lower CF and whole-body moisture content (*p* < 0.05) (Table 2 and Table 3).

### 3.3. P absorption and Metabolism

**Figure 2:**
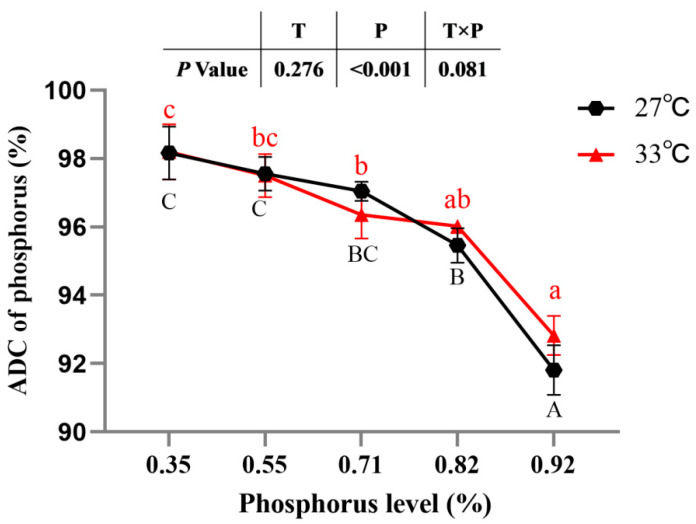
Apparent digestibility coefficient (ADC) of diets with different available phosphorus levels in spotted seabass and raised at 27 °C and 33 °C. Significant differences are indicated by capital letters at 27 °C and lowercase letters at 33 °C (*p* < 0.05).

The ADC of P was not significantly affected by temperature or the interaction between water temperature and available P levels (*p >* 0.05). However, the ADC of P of spotted seabass was significantly affected by the available P levels. The ADC of P of spotted seabass fed diets with 0.35% and 0.55% available P levels was significantly higher than that of spotted seabass fed the diet with 0.92% available P level at 27 °C and 33 °C (*p* < 0.05) (Figure 2).

**Figure 3:**
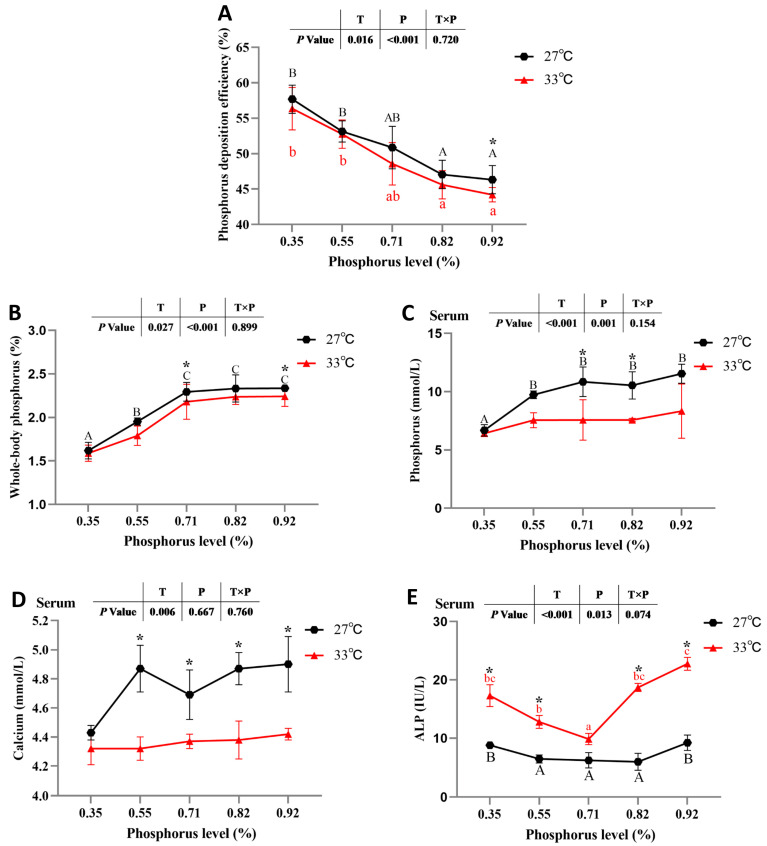
(**A**) Phosphorus deposition efficiency, (**B**) whole-body phosphorus content, (**C**) serum phosphorus content, (**D**) serum calcium content and (**E**) serum alkaline phosphatase (ALP) activity of spotted seabass fed diets with different available phosphorus levels and raised at 27 °C and 33 °C. Significant differences are indicated by capital letters at 27 °C and lowercase letters at 33 °C (*p* < 0.05). At the same phosphorus level, there was a significant difference between 27 °C and 33 °C, as indicated by an asterisk (*) (*p* < 0.05).

There were no significant interactions between water temperature and available P levels on whole-body P deposition efficiency, whole-body or serum P contents, serum calcium content or serum ALP activity (*p* > 0.05). Regardless of available P levels, spotted seabass raised at 33 °C had significantly lower whole-body P deposition efficiency, whole-body and serum P, and serum calcium contents and significantly higher serum ALP activity than those of spotted seabass raised at 27 °C (*p* < 0.05) (Figure 3).

The whole-body P deposition efficiency of spotted seabass fed diets with 0.35% and 0.55% available P levels was significantly higher than that of spotted seabass fed diets with 0.82% and 0.92% available P levels at 27 °C and 33 °C (*p* < 0.05). Spotted seabass fed diets with 0.35% available P level had the lowest whole-body P and serum P contents among dietary treatments at 27 °C (*p* < 0.05), whereas there were no significant differences in whole-body P or serum P contents among dietary treatments at 33 °C. The serum ALP activity of spotted seabass fed diets with 0.35% and 0.92% available P levels was significantly higher than that of spotted seabass fed diets with 0.55%, 0.71% and 0.82% available P levels at 27 °C (*p* < 0.05), and spotted seabass fed diets with 0.71% had the lowest serum ALP activity among dietary treatments at 33 °C (*p* < 0.05). Irrespective of water temperature, there was no significant effect of available P levels on the serum calcium content of spotted seabass (*p* > 0.05) (Figure 3).

**Figure 4:**
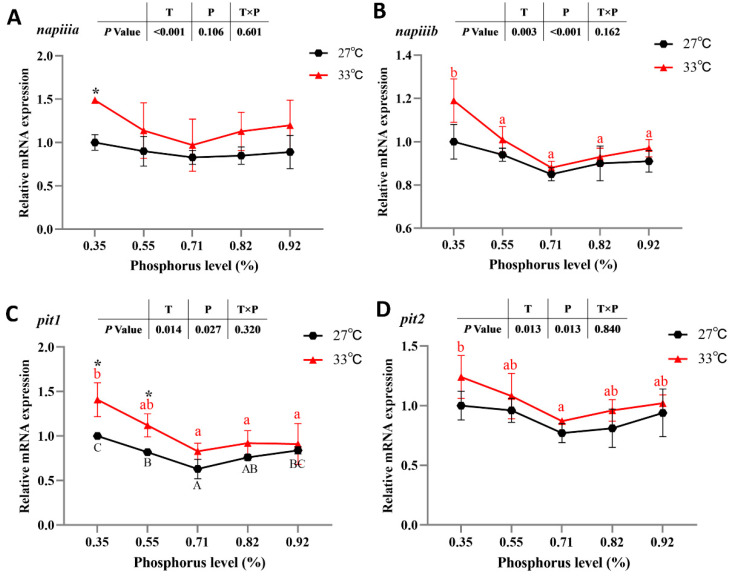
Intestinal phosphorus transport-related genes ((**A**) *napiiia*; (**B**) *napiiib*; (**C**) *pit1*; (**D**) *pit2*) mRNA expression of spotted seabass fed diets with different available phosphorus levels and raised at 27 °C and 33 °C. Significant differences are indicated by capital letters at 27 °C and lowercase letters at 33 °C (*p* < 0.05). At the same phosphorus level, there was a significant difference between 27 °C and 33 °C, as indicated by an asterisk (*) (*p* < 0.05).

Irrespective of available P levels, spotted seabass raised at 33 °C showed significantly higher relative mRNA expression of intestinal P transport-related genes (*napiiia*, *napiiib*, *pit1* and *pit2*) than that of spotted seabass raised at 27 °C (*p* < 0.05). The relative mRNA expression of *pit1* in spotted seabass fed the diet with 0.35% available P level was significantly higher than that of spotted seabass fed diets with 0.55%, 0.71% and 0.82% available P levels at 27 °C (*p* < 0.05). The relative mRNA expression of *napiiib*, *pit1* and *pit2* in spotted seabass fed the diet with 0.35% available P level was significantly higher than that of spotted seabass fed the diet with 0.71% available P level at 33 °C (*p* < 0.05) (Figure 4).

Phosphorus deposition efficiency = 100 × (final body weight × final fish whole-body phosphorus content − initial body weight × initial fish whole-body phosphorus content)/(feed intake × phosphorus content in feed).

### 3.4. Lipid Metabolism and Liver Health Indices

**Figure 5:**
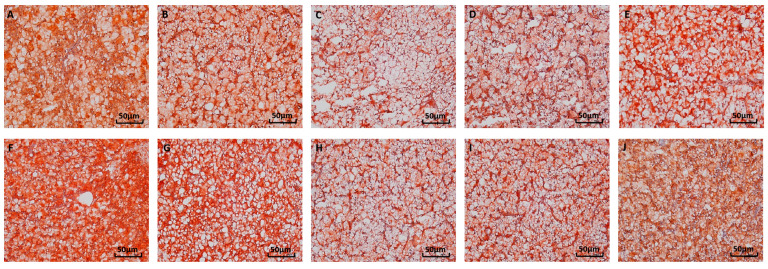
Liver morphology of spotted seabass fed diets with different available phosphorus levels and raised at 27 °C and 33 °C (oil red O staining, magnification of 200×). (**A**) 27 °C/0.35% P, (**B**) 27 °C/0.55% P, (**C**) 27 °C/0.71% P, (**D**) 27 °C/0.82% P, (**E**) 27 °C/0.92% P, (**F**) 33 °C/0.35% P, (**G**) 33 °C/0.55% P, (**H**) 33 °C/0.71% P, (**I**) 33 °C/0.82% P, (**J**) 33 °C/0.92% P.

**Figure 6:**
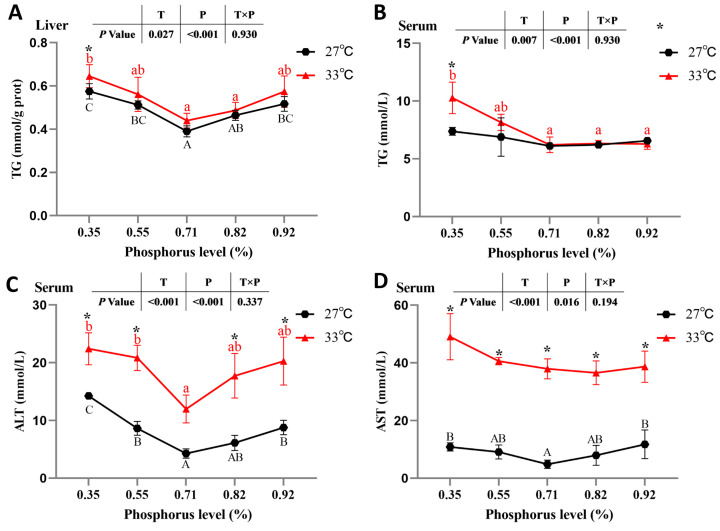
(**A**) Liver triacylglycerols (TG) content, (**B**) serum TG content, (**C**,**D**) serum glutamic pyruvic transaminase (ALT) and glutamic oxaloacetic transaminase (AST) activities of spotted seabass fed diets with different available phosphorus levels and raised at 27 °C and 33 °C. Significant differences are indicated by capital letters at 27 °C and lowercase letters at 33 °C (*p* < 0.05). At the same phosphorus level, there was a significant difference between 27 °C and 33 °C, as indicated by an asterisk (*) (*p* < 0.05).

The results showed no significant interaction between water temperature and available P levels on hepatic and serum TG contents, serum ALT and AST activities, or the expression of hepatic lipid-metabolism-related genes (*p >* 0.05) (Figure 5 and Figure 6, Table 4). The results of oil red O staining showed that the highest number of hepatic lipid droplets was found in spotted seabass fed the diet with 0.35% available P level at 33 °C, and the lowest number of hepatic lipid droplets was found in spotted seabass fed the diet with 0.71% available P level at 27 °C (Figure 5). Irrespective of available P levels, spotted seabass raised at 33 °C exhibited significantly higher hepatic and serum TG contents, as well as serum ALT and AST activities, than those of spotted seabass raised at 27 °C (*p* < 0.05) (Figure 6).

The hepatic TG content of spotted seabass fed the diet with 0.35% available P level was significantly higher than that of spotted seabass fed diets with 0.71% and 0.82% available P levels at 27 °C and 33 °C (*p* < 0.05). The serum TG content of spotted seabass fed the diet with 0.35% available P level was significantly higher than that of spotted seabass fed diets with 0.71%, 0.82% and 0.92% available P levels at 33 °C (*p* < 0.05). The serum ALT activity of spotted seabass fed diets with 0.35%, 0.55% and 0.92% available P levels was significantly higher than that of spotted seabass fed the diet with 0.71% available P level at 27 °C (*p* < 0.05), and the serum ALT activity in spotted seabass fed diets with 0.35% and 0.55% available P levels was significantly higher than that of spotted seabass fed the diet with 0.71% available P level at 33 °C (*p* < 0.05). In addition, the serum AST activity in spotted seabass fed diets with 0.35% and 0.92% available P levels was significantly higher than that of spotted seabass fed the diet with 0.71% available P level at 27 °C (*p* < 0.05) (Figure 6).

The expression of *fas* and *srebp-1* was significantly increased, and the expression of *atgl* was significantly decreased in spotted seabass raised at 33 °C compared to those of spotted seabass raised at 27 °C (*p* < 0.05). Regardless of water temperature, compared to spotted seabass fed the diet with 0.71% available P level, spotted seabass fed diets with 0.35%, 0.55% and 0.92% available P levels had significantly higher expressions of *fas* and *srebp-1* and significantly lower expressions of *cpt-1* (*p* < 0.05). In addition, compared to spotted seabass fed diets with 0.35% and 0.55% available P levels, the expression of *atgl* was significantly higher in spotted seabass fed the diet with 0.71% available P level (*p* < 0.05) (Table 4).

### 3.5. Serum Antioxidant Status

There were no significant interactions between water temperature and available P levels on serum antioxidant indices. Irrespective of available P levels, spotted seabass raised at 33 °C showed significantly increased serum T-AOC, content of MDA, and activities of CAT, SOD and GSH-PX compared to those of spotted seabass raised at 27 °C (*p* < 0.05). Regardless of water temperature, compared to spotted seabass fed diets with 0.35%, 0.55% and 0.92% available P levels, spotted seabass fed the diet with 0.71% available P level had significantly higher activity of SOD and T-AOC and significantly lower content of MDA (*p* < 0.05). In addition, the lowest activity of GSH-PX was found in spotted seabass fed the diet with 0.35% available P level among dietary treatments. Regardless of water temperature, available P levels had no significant effect on the serum CAT activity of spotted seabass (*p* > 0.05) (Table 5).

### 3.6. Intestinal Microbiota Composition

**Figure 7:**
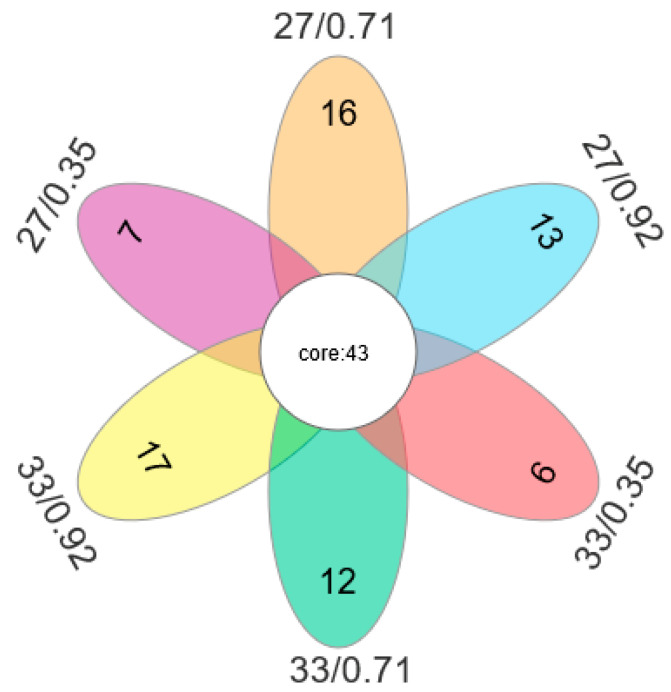
Venn diagrams were used to compare OTUs of intestinal bacterial communities of spotted seabass fed diets with different available phosphorus levels and raised at 27 °C and 33 °C.

Spotted seabass fed diets with relatively low (0.35%), medium (0.71%), and high (0.92%) available P levels were selected for intestinal microbiota sequencing at 27 °C and 33 °C. For each of the 18 samples, quality screening yielded at least 112,898 excellent sequences (clean tags) (three replicates per group). A total of 342 OTUs were observed in all samples, and the OTUs specific to each group were 16, 27 °C/0.35% P; 13, 27 °C/0.71% P; 6, 27 °C/0.92% P; 12, 33 °C/0.35% P; 17, 33 °C/0.71% P; 7, 33 °C/0.92% P, respectively (Figure 7).

**Figure 8:**
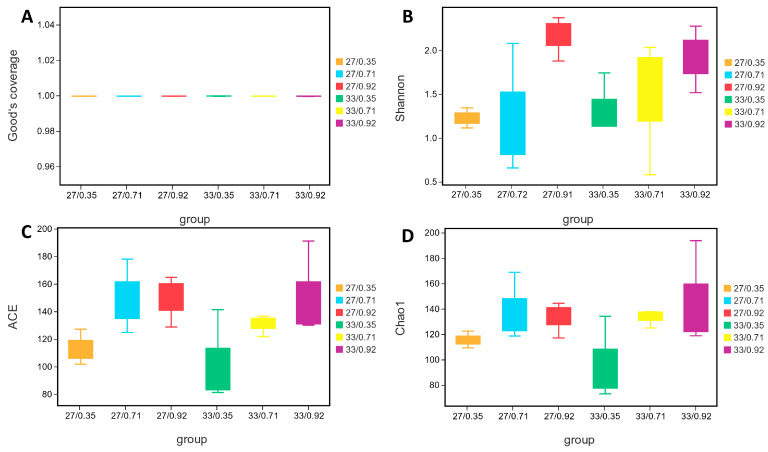
Intestinal microbial alpha diversity index of spotted seabass fed diets with different phosphorus available levels and raised at 27 °C and 33 °C. (**A**) good’s coverage (**B**) Shannon index, (**C**) ACE index, (**D**) chao1 index.

The results revealed that sample coverage was greater than 98% across all groups. Significant interactions were found between water temperature and available P level on the abundances of *Bacillus* and *Lactococcus*. Simple effects analysis showed that the abundance of *Bacillus* showed the highest value (27.64%) in spotted seabass fed the diet with 0.92% available P level at 33 °C, and the abundance of *Lactococcus* showed the highest value (26.02%) in spotted seabass fed the diet with 0.71% available P level at 27 °C among the 10 treatments (Table 6). Regardless of available P levels, spotted seabass raised at 33 °C showed higher microbial α-diversity indices Shannon, ACE, and Chao1 than those of spotted seabass raised at 27 °C. Regardless of water temperature, the α-diversity indices Shannon, ACE, and Chao1 of spotted seabass increased as available P levels in the diet increased from 0.35% to 0.71% (Figure 8).

**Figure 9:**
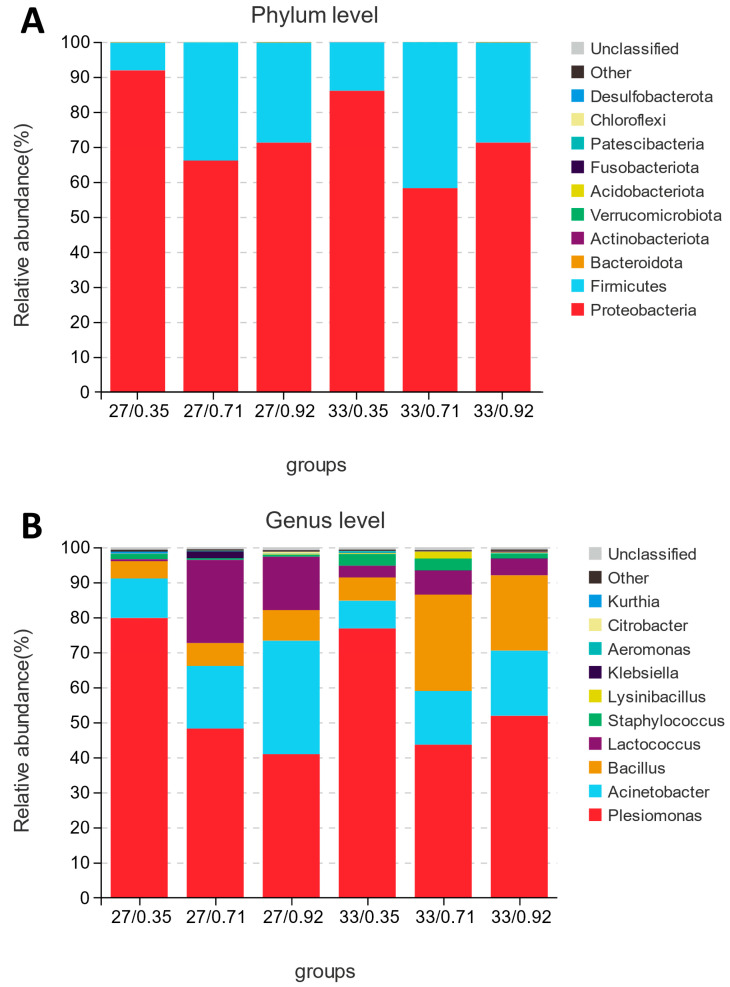
Intestinal microbial composition of spotted seabass fed diets with different available phosphorus levels and raised at 27 °C and 33 °C. (**A**) phylum level, (**B**) genus level.

Intestinal microbial species composition at the phylum level showed a significant increase (*p* < 0.05) in the abundance of Firmicutes (9.97% to 37.91%) and a significant decrease (*p* < 0.05) in the abundance of Proteobacteria (89.71% to 61.09%) in spotted seabass as available P levels of the diet increased from 0.35% to 0.71% (Figure 9). Water temperature had no significant effect on species composition at the phylum level.

At the genus level, regardless of available P levels, spotted seabass raised at 33 °C significantly (*p* < 0.05) increased the abundance of *Bacillus* (6.78% to 18.18%) and decreased the abundance of *Lactococcus* (15.49% to 3.52%) when compared to spotted seabass raised at 27 °C. Regardless of water temperature, there was a significant increase (*p* < 0.05) in the abundance of *Bacillus* and *Lactococcus* (5.93% to 13.92%, 2.43% to 15.36%), as well as a significant decrease (*p* < 0.05) in the abundance of *Plesiomonas* (77.29% to 45.22%), in spotted seabass as the available P levels of the diet increased from 0.35% to 0.71%.

## 4. Discussion

In the present study, FBW and WG of spotted seabass were significantly increased as the available P levels in the diet increased from 0.35% to 0.71%, which revealed that 0.35% available P in the diet was insufficient to meet the needs of spotted seabass for growth, necessitating the use of additional P supplements. Similar results were also observed in crucian carp (*Carassius auratus*) [30], grass carp (*Ctenopharyngodon idella*) [31], and rainbow trout (*Oncorhynchus mykiss*) [32]. In addition, a study on Songpu Mirror Carp (*Cyprinus carpio Songpu*) found that P supplementation in the diet promoted intestinal health and feed utilization [33]. In this study, it was also found that an available P level of 0.71% diet significantly increased the feed utilization of spotted seabass compared to a basal diet with an available P level of 0.35%, which was probably responsible for the improvement in fish growth. Afterward, with the increase in available P levels from 0.71% to 0.92% in the diet, the growth rate of spotted seabass reached a plateau or even decreased. This could be due to the fact that excessive P intake inhibited the absorption and metabolism of other elements (e.g., Mg, Zn, Fe, etc.), which ultimately restrained growth; a similar result was also reported in juvenile silver perch (*Bidyanus bidyanus*) [34].

Water temperature affects the growth and metabolism of fish throughout their growth period [35]. Spotted seabass is a typical temperate fish that prefers water temperatures ranging from 18 to 27 °C, with 33 °C being considered a high water temperature for this species [23,36]. In this study, according to a second-order polynomial regression analysis of weight gain rate on dietary available P levels, the optimal dietary available P level for spotted seabass was 0.72% and 0.78% at 27 °C and 33 °C, respectively, which indicated that water temperature affected the dietary P requirement of spotted seabass. P is an essential component of energy substances like ATP, and compared to 27 °C, spotted seabass requires more P at 33 °C. This may be attributed to the fact that high temperatures cause an acceleration in the metabolic rate of fish, resulting in increased energy consumption to adapt to the high-temperature environment [37]. In addition, the WG of spotted seabass raised at 33 °C was significantly lower than that of 27 °C. In the studies of Nile tilapia (*Oreochromis niloticus*) [38], juvenile spotted wolffish (*Anarhichas minor*) [12] and juvenile sablefish (*Anoplopoma fimbria*) [39], high temperatures significantly inhibited the growth rate, which could be due to the reduction in nutrients and energy used for growth and development of fish in a high-temperature environment.

The utilization rate of organic P In diet ingredients is relatively lower, with the apparent digestibility of P in fish meal generally ranging from 20% to 70% [40]. In this experiment, compared to other research results, the apparent digestibility of dietary P was higher. This could be due to the use of boneless fish meal in the diet, which has a lower organic P content than regular fish meal. Fish must constantly and efficiently absorb, store and turnover P to ensure a normal metabolism and growth of the organism [41]. The results of the present study showed that whole-body P content and serum P content increased significantly as the levels of available P increased from 0.35% to 0.71% in the diet. The high P contents in tissues and blood allow fish to mobilize much P to participate in the formation of bones and key metabolic components, leading to a good growth performance [42,43]. In addition, excessive P intake in this study did not result in higher P deposition in the blood and whole body. This is because there is a limit to the absorption of P by fish, and any P in the feed that cannot be absorbed by the fish is excreted in the form of feces and urine. Studies in rainbow trout have also shown that high P increases fecal and non-fecal P excretion [44].

The regulation of Na-Pi expression in the intestines is a key pathway for maintaining P homeostasis in the body. The main pathway of intestinal P absorption is carried out by Na-Pi transporters, which mainly includes three subtypes: Napi-iia, Napi-iib and Napi-iic (PiT1 and PiT2) [45]. In this study, the highest expression levels of intestinal P transport-related genes were found in spotted seabass fed the diet with an available P level of 0.35%. P deficiency has also been found, in other studies, to cause the upregulation of genes related to P transport [46]. Combining the results of the ADC of P and whole-body P deposition efficiency, it is hypothesized that this can be a regulatory mechanism for the perch to adapt to the P-deficient environment by improving the absorption, utilization and deposition of P.

Fluctuations in water temperature affect the uptake and metabolic processes of aquatic animals, which can respond to environmental changes through regulation in physiology and biochemical metabolism [47]. In the present study, the P content in the serum and the rate of P deposition in the whole-body were significantly lower in spotted seabass raised at 33 °C compared to those of fish raised at 27 °C. Wang, Li, Lu, Wang, Ma, Song and Zhang [14] also found that a high temperature reduced iron deposition in spotted seabass and thus affected iron homeostasis. This could be due to the fact that a high temperature caused metabolic disorders in the organism, which required more nutrients to alleviate the metabolic disorders, resulting in lower nutrient deposition [48]. In addition, the expression level of intestinal P transport-related genes and the activity of serum ALP were increased in spotted seabass raised at 33 °C. The increase in water temperature enhances the activity of enzymes in fish, and the increase in ALP activity can promote osteoclasts to induce P to enter the bloodstream and participate in the metabolism of the organism [49]. Spotted seabass was expected to maintain its metabolism by increasing P uptake in response to the increased nutrient consumption caused by high temperatures. Therefore, spotted seabass probably needed more P to maintain the physiological homeostasis of the organism under high-temperature conditions.

P plays an important role in lipid metabolism, including the synthesis of phospholipids, and phosphorylation modification [50]. Avila et al. [51] noted that P deficiency promoted lipid deposition, causing the protein to be used as an energy source rather than for growth. In the present study, spotted seabass fed the low-P diet had the highest whole-body crude lipid and TG contents. As dietary available P levels increased, serum and liver TG content and whole-body crude lipid content of spotted seabass were significantly decreased, and crude protein content was significantly increased. These results suggested that P supplementation reduced body lipid content and improved protein deposition. In the process of lipid metabolism, CPT-1 and ATGL separately regulate lipolytic processes via fatty acid β-oxidation and triglyceride hydrolysis [52,53]. SREBP-1c is a key regulator of hepatic lipid metabolism, regulating the transcription of nearly all hepatic triglyceride and fatty acid synthesis genes [54]. Wueest et al. [55] discovered that FAS overexpression resulted in abnormal lipid deposition. Thus, the results of lipid-metabolism-related gene expression in the present study indicated that 0.71–0.82% P supplementation in the diet could reduce lipid synthesis and improve lipid catabolism, which led to better growth and less lipid deposition in spotted seabass.

Increasing the water temperature is also known to directly impact ectotherms’ energy needs, which, in turn, affects their lipid metabolism and usage of lipid depots [56]. Studies on juvenile turbot found that elevated water temperatures led to lipid accumulation, reduced hepatic β-oxidation, and decreased lipolytic enzyme activity, disrupting the balance of lipid metabolism [57,58]. Spotted seabass raised at 33 °C in the current study displayed more lipid deposition than spotted seabass raised at 27 °C, along with increased expression levels of the lipid-synthesis-related genes *fas* and *srebp-1,* and decreased the expression levels of the lipolysis-related gene *atgl*, which was consistent with the findings of previous studies on Atlantic salmon (*Salmo salar* L.) [59] and Pacu (*Piaractus mesopotamicus*) [60]. These results suggested that higher water temperatures triggered a disturbance of lipid deposition and lipid metabolism in spotted seabass, which could be responsible for the poor growth of spotted seabass raised at 33 °C. In addition, the disturbances of lipid deposition and lipid metabolism caused by high temperature were weakened in fish fed a 0.71–0.82% P supplementation diet. This indicated that supplementing the diet with appropriate levels of P could alleviate, to some extent, the disturbance of lipid metabolism and abnormal lipid deposition caused by high temperature.

Dietary P level is known to influence the antioxidant status and immunity of fish [61]. According to studies, P deficiency reduced antioxidant enzyme activities and GSH content, and adding appropriate levels of P to the diet could improve SOD activity and reduce MDA content [62,63]. The activities of GSH-PX, SOD and T-AOC in the serum of spotted seabass increased with the increase in available P levels from 0.35% to 0.71%, while the content of MDA decreased. The antioxidant enzymes CAT and SOD are thought to be the first line of defense against excessive reactive oxygen species (ROS) [64]. T-AOC and GSH-PX are found to be important in the scavenge of superoxide anion, hydrogen peroxide, and lipid hydroperoxides, which can help to reduce oxidative damage under stress [65]. As a result, 0.71–0.82% P supplementation in the diet could improve the ability of enzymes to act as antioxidants and scavenge free radicals. A study reported that P promoted glutathione synthesis by providing ATP required for glutathione synthesis [66]. Xilan et al. [67] indicated that P could enhance CAT activity by increasing NADPH content. However, the exact mechanisms of how P enhanced antioxidant capacity still need to be investigated in more depth.

The antioxidant status of fish is directly affected by changes in environmental temperature [68]. Increasing the antioxidant enzyme activity contributes to the resistance of fish to various stressful environments, such as high temperature and excessive culture density [69]. In the present study, serum MDA levels, T-AOC, and the activities of CAT, SOD, and GSH-Px were significantly increased in spotted seabass raised at 33 °C compared to those raised at 27 °C. MDA was a by-product of lipid peroxidation induced by oxidative stress [70], and the level of lipid peroxidation significantly increased under high-temperature stress, which indicated that the high temperature exacerbated oxidative damage in spotted seabass. Also, high temperatures increased the activities of antioxidant enzymes in spotted seabass, which may be a result of the organism’s response to elevated ROS levels by mobilizing enzymatic antioxidants with the aim of mitigating the adverse effects of ROS. This adaptive mechanism was also reported in other fish, such as yellow catfish (*Pelteobagrus fulvidraco*) [71] and Golden pompano (*Trachinotus blochii*) [72].

Intestinal microbiota play an important role in the health, immunity, metabolism, development, and reproduction of fish [73]. Dietary nutrition is a critical factor in determining the makeup of the trillions of bacteria that reside in the intestine [74]. Labaw et al. [75] demonstrated that P, as a component of nucleic acid and other biochemical molecules, played a vital role in microorganism growth [76]. In a rat study, a P-rich diet was found to improve colonization resistance to intestinal pathogens and to promote lactic acid bacteria in the digestive tract [77]. Under the present experimental conditions, P supplementation in the diet increased the diversity of intestinal microbiota and improved the microbial composition in spotted seabass. Compared with the low-P diet group, P supplementation in the diet decreased the abundance of *Plesiomonas* and increased the abundance of *L. lactis* and *Bacillus* in the intestine of spotted seabass. *Plesiomonas* is proposed as a possible opportunistic pathogen in fish intestine that can damage fish health by causing enteritis and indigestion [78]. *Bacillus*, a well-known potential probiotic, has been shown to improve the intestinal microbiota by increasing the number of beneficial microorganisms while decreasing the number of pathogens [79]. In addition, *Lactococcus* is also known to improve fish growth performance and inhibit the growth of pathogenic bacteria [80,81]. Therefore, dietary P supplementation could improve the composition of the intestinal microbiota.

The intestinal microbiota form a mutually beneficial symbiotic relationship with the host, where microorganisms can play beneficial roles, such as maintaining a normal immune system, preventing pathogen colonization, and facilitating the digestion and absorption of nutrients [82]. The increased abundance of *Lactococcus* and *Bacillus* in the intestine of spotted seabass fed diets with available P levels of 0.71% versus 0.92% resulted in a better utilization of nutrients and enhanced absorption and metabolism, which led to better growth performance. In addition, *Bacillus* has been reported to have a potential function in reducing oxidative stress [83]. Therefore, the increased abundance of *Bacillus* could contribute to improving the antioxidant capacity of the organism. Therefore, the supplementation of a diet with 0.71–0.92% P could increase the abundance of potential probiotics and decrease the abundance of potentially pathogenic bacteria, which, in turn, played a positive and beneficial role in the growth and metabolism of the organism and protection against tissue damage.

The balance of the intestinal microbiota is susceptible to various factors in the external water environment [84]. On the one hand, changes in water temperature could affect the abundance and structure of the intestinal microbiota [85]. In a study of Atlantic salmon, elevated water temperatures resulted in a shift in its intestinal microbiota from generally beneficial lactic acid bacteria to the potentially pathogenic genus *Vibrio* [86]. In the present study, a high temperature did not significantly affect microbial diversity but significantly reduced the abundance of *Lactococcus*, and similar results were found in Yellowtail amberjack (*Seriola lalandi*) [87]. *Lactococcus* has been shown to improve growth performance and nutrient uptake metabolism, resist pathogen colonization, and mitigate intestinal tissue damage [88]. Therefore, the negative effects caused by high temperature in this experiment could be related to the decrease in the relative abundance of *Lactococcus* in the intestine.

On the other hand, the plasticity of the intestinal microbiota ensures rapid host adaptation to environmental changes [89]. The intestinal microbiota is shown to play an important role in counteracting the negative effects of heat stress [90]. In the present study, the high temperature also increased the abundance of *Bacillus* in the intestine of spotted seabass. Moore et al. [91] indicated that *Bacillus* could protect animals from heat stress by improving gut integrity and gut microbiota, allowing for a faster recovery. The potential function of *Bacillus* to reduce oxidative stress was also mentioned above. Hence, the increased abundance of *Bacillus* in the intestine could be an evolutionary strategy for the spotted seabass in response to high-temperature stress, and this adaptive mechanism allowed for greater resilience and buffering capacity.

This experiment comprehensively investigated the effects of available P levels on the physiological metabolism of spotted seabass at moderate and high temperatures, and obtained the requirements at the corresponding temperatures. However, there are some potential limitations of this experiment. The high water temperature treatment may cause irreversible damage to the fish at the beginning of the experiment, affecting fish feeding and thus the results of subsequent experiments. In further experiments, setting more temperature levels for comparison could further strengthen the reliability of the conclusions of this experiment.

## 5. Conclusions

A high water temperature (33 °C) significantly decreased growth performance and caused oxidative stress and abnormal lipid deposition in spotted seabass compared to a moderate water temperature (27 °C). Meanwhile, the high temperature increased the P uptake, antioxidant enzyme activity and the abundance of *Bacillus* in the intestine of spotted seabass to adapt to the high-temperature environment. Insufficient or excessive dietary P was detrimental to the growth of spotted seabass. Dietary supplementation with 0.71–0.82% available P improved the growth performance, antioxidant capacity and microbial composition of spotted seabass, and alleviated the disturbance of lipid metabolism and abnormal lipid deposition caused by a high temperature or low-P diet. The available P requirement for the diet of spotted seabass at 27 °C and 33 °C was 0.72% and 0.78%, respectively.

## Figures and Tables

**Table 1:** Growth performance and feed utilization of spotted seabass fed diets with different available phosphorus levels and raised at 27 °C and 33 °C.

		FBW ^a^	WG ^b^	SR ^c^	FR ^d^	FCR ^e^
27 °C	0.35%	38.50 ± 0.66 ^A^	972.42 ± 16.01 ^A^	91.11 ± 5.09	2.64 ± 0.29	1.29 ± 0.03
	0.55%	56.10 ± 1.23 ^BC^	1489.44 ± 38.15 ^BC^	96.67 ± 0.00	2.60 ± 0.42	1.22 ± 0.04
	0.71%	69.78 ± 2.29 ^E^	1883.95 ± 25.27 ^E^	96.67 ± 0.00	2.49 ± 0.28	0.93 ± 0.11
	0.82%	66.13 ± 1.29 ^DE^	1782.44 ± 49.27 ^DE^	96.67 ± 0.00	2.55 ± 0.36	1.03 ± 0.05
	0.92%	52.65 ± 0.19 ^BC^	1405.81 ± 1.53 ^BC^	100.00 ± 0.00	2.71 ± 0.18	1.06 ± 0.11
33 °C	0.35%	38.16 ± 1.35 ^A^	969.92 ± 31.01 ^A^	85.56 ± 8.39	3.02 ± 0.10	1.46 ± 0.11
	0.55%	51.78 ± 1.49 ^B^	1376.43 ± 32.69 ^B^	100.00 ± 0.00	2.88 ± 0.40	1.30 ± 0.02
	0.71%	64.02 ± 0.91 ^D^	1727.49 ± 37.09 ^D^	98.89 ± 1.92	2.50 ± 0.14	1.07 ± 0.04
	0.82%	65.63 ± 2.53 ^DE^	1759.04 ± 64.23 ^D^	100.00 ± 0.00	2.62 ± 0.10	1.08 ± 0.06
	0.92%	54.63 ± 2.56 ^C^	1515.15 ± 68.19 ^C^	100.00 ± 0.00	3.12 ± 0.14	1.31 ± 0.06
Temperature						
27 °C		56.63±11.47 *	1506.81 ± 332.99 *	96.22 ± 3.53	2.60 ± 0.26	1.11 ± 0.15
33 °C		54.49±10.32	1452.74 ± 288.55	96.89 ± 6.72	2.83 ± 0.31 *	1.24 ± 0.16 *
Phosphorus						
0.35%		38.33±0.97 ^a^	971.17 ± 22.12 ^a^	88.34 ± 6.91 ^a^	2.83 ± 0.29	1.38 ± 0.11 ^c^
0.55%		53.94±2.67 ^b^	1432.94 ± 69.58 ^b^	98.33 ± 1.82 ^b^	2.74 ± 0.39	1.26 ± 0.05 ^bc^
0.71%		66.89±3.52 ^c^	1805.72 ± 90.27 ^c^	97.78 ± 1.7 ^b^	2.50 ± 0.19	1.00 ± 0.10 ^a^
0.82%		65.93±1.58 ^c^	1773.08 ± 49.09 ^c^	98.34 ± 1.82 ^b^	2.58 ± 0.24	1.06 ± 0.05 ^a^
0.92%		54.62±2.69 ^b^	1460.48 ± 73.81 ^b^	100.00 ± 0.00 ^b^	2.92 ± 0.26	1.18 ± 0.15 ^b^
*p* value						
Temperature		0.025	0.023	0.572	0.032	<0.001
Phosphorus		<0.001	<0.001	<0.001	0.078	<0.001
Interaction		0.010	0.007	0.120	0.618	0.141

Significant differences (*p* < 0.05) are highlighted in a column with different symbols (capital letters, simple effects analysis; “*”, water temperature; lowercase letters, phosphorus levels). ^a^ Final body weight (g). ^b^ Weight gain (%) = (final body weight − initial body weight)/initial body weight × 100. ^c^ Survival rate (%) = final fish number/initial fish number × 100. ^d^ Feeding rate (%/day) = dry feed intake/(initial body weight/2 + final body weight/2)/feeding days × 100. ^e^ Feed conversion rate = dry feed fed/wet weight gain.

**Table 2:** Morphological parameters of spotted seabass fed diets with different available phosphorus levels and raised at 27 °C and 33 °C.

		HSI ^a^	VSI ^b^	CF ^c^	IFR ^d^
27 °C	0.35%	1.86 ± 0.31	12.57 ± 0.51	2.05 ± 0.09	6.35 ± 0.41 ^A^
	0.55%	1.87 ± 0.23	12.23 ± 0.79	2.01 ± 0.07	6.35 ± 0.35 ^A^
	0.71%	1.74 ± 0.17	12.72 ± 1.11	1.93 ± 0.09	6.24 ± 0.31 ^A^
	0.82%	1.78 ± 0.19	11.64 ± 0.33	1.89 ± 0.07	6.24 ± 0.28 ^A^
	0.92%	1.92 ± 0.16	13.06 ± 0.22	1.81 ± 0.06	6.53 ± 0.09 ^A^
33 °C	0.35%	2.68 ± 0.28	15.96 ± 1.40	2.32 ± 0.02	8.81 ± 0.17 ^C^
	0.55%	1.94 ± 0.20	15.52 ± 0.32	2.10 ± 0.05	8.76 ± 0.14 ^C^
	0.71%	1.95 ± 0.22	16.03 ± 0.79	1.99 ± 0.11	7.86 ± 0.08 ^B^
	0.82%	2.01 ± 0.20	15.79 ± 0.89	2.02 ± 0.06	8.82 ± 0.16 ^C^
	0.92%	1.99 ± 0.05	15.56 ± 0.43	2.04 ± 0.05	8.06 ± 0.17 ^BC^
Temperature					
27 °C		1.83 ± 0.19	12.44 ± 0.76	1.94 ± 0.11	6.34 ± 0.28
33 °C		2.11 ± 0.33 *	15.77 ± 0.75 *	2.09 ± 0.14 *	8.46 ± 0.49 *
Phosphorus					
0.35%		2.27 ± 0.52 ^b^	14.26 ± 2.08	2.19 ± 0.16 ^c^	7.58 ± 1.02 ^b^
0.55%		1.90 ± 0.19 ^a^	13.88 ± 1.88	2.06 ± 0.07 ^b^	7.55 ± 0.86 ^b^
0.71%		1.84 ± 0.20 ^a^	14.37 ± 2.01	1.96 ± 0.09 ^ab^	7.05 ± 1.39 ^a^
0.82%		1.90 ± 0.22 ^a^	13.71 ± 2.35	1.95 ± 0.09 ^ab^	7.53 ± 1.43 ^ab^
0.92%		2.01 ± 0.11 ^ab^	14.31 ± 1.40	1.92 ± 0.13 ^a^	7.29 ± 1.26 ^ab^
*p* value					
Temperature		0.002	<0.001	<0.001	<0.001
Phosphorus		0.034	0.500	<0.001	0.021
Interaction		0.217	0.505	0.060	0.009

Significant differences (*p* < 0.05) are highlighted in a column with different symbols (capital letters, simple effects analysis; “*”, water temperature; lowercase letters, phosphorus levels). ^a^ Hepatosomatic index (HSI, %) = liver weight/final body weight × 100. ^b^ Viscerosomatic index (VSI, %) = visceral weight/final body weight × 100. ^c^ Condition factor (CF, g/cm^3^) = final body weight/final body length^3^ × 100. ^d^ Intraperitoneal fat rate (IFR, %) = intraperitoneal fat weight/final body weight × 100.

**Table 3:** Body compositions (%) of spotted seabass fed diets with different available phosphorus levels and raised at 27 °C and 33 °C.

		Moisture	Ash	Lipid	Protein
27 °C	0.35%	68.76 ± 0.54	2.38 ± 0.06	11.17 ± 0.32	15.90 ± 0.30
	0.55%	67.96 ± 0.34	3.16 ± 0.08	10.35 ± 0.30	16.51 ± 0.37
	0.71%	67.28 ± 0.79	3.70 ± 0.28	9.85 ± 0.89	17.67 ± 1.15
	0.82%	67.54 ± 0.84	3.71 ± 0.17	9.91 ± 0.69	17.04 ± 1.02
	0.92%	66.92 ± 0.24	4.05 ± 0.17	10.74 ± 0.66	16.92 ± 0.78
33 °C	0.35%	69.55 ± 0.23	2.49 ± 0.12	12.18 ± 0.36	14.41 ± 0.49
	0.55%	69.17 ± 0.77	3.57 ± 0.27	11.77 ± 0.59	14.66 ± 0.44
	0.71%	68.41 ± 0.31	3.74 ± 0.28	11.51 ± 0.91	15.75 ± 1.07
	0.82%	68.09 ± 0.36	3.94 ± 0.40	11.50 ± 0.66	15.44 ± 0.77
	0.92%	67.13 ± 0.86	4.47 ± 0.25	12.05 ± 0.67	15.22 ± 0.95
Temperature					
27 °C		67.69 ± 0.83	3.40 ± 0.62	10.41 ± 0.73	16.81 ± 0.91 *
33 °C		68.47 ± 1.01 *	3.64 ± 0.71 *	11.80 ± 0.63 *	15.10 ± 0.84
Phosphorus					
0.35%		69.16 ± 0.57 ^c^	2.43 ± 0.11 ^a^	11.67 ± 0.63 ^b^	15.16 ± 0.75 ^a^
0.55%		68.57 ± 0.85 ^bc^	3.36 ± 0.29 ^b^	11.06 ± 0.88 ^ab^	15.59 ± 1.21 ^ab^
0.71%		67.85 ± 0.82 ^ab^	3.72 ± 0.25 ^bc^	10.68 ± 1.21 ^a^	16.71 ± 1.44 ^b^
0.82%		67.82 ± 0.65 ^ab^	3.83 ± 0.30 ^c^	10.71 ± 1.06 ^a^	16.24 ± 1.16 ^b^
0.92%		67.03 ± 0.58 ^a^	4.26 ± 0.29 ^d^	11.39 ± 0.93 ^ab^	16.07 ± 1.24 ^ab^
*p* value					
Temperature		0.002	0.010	<0.001	<0.001
Phosphorus		<0.001	<0.001	0.039	0.033
Interaction		0.564	0.538	0.909	0.984

Significant differences (*p* < 0.05) are highlighted in a column with different symbols (“*”, water temperature; lowercase letters, phosphorus levels).

**Table 4:** The mRNA expression of lipid-metabolism-related genes in the liver of spotted seabass fed diets with different available phosphorus levels and raised at 27 °C and 33 °C.

		Lipogenesis		Lipolysis		
		fas	srebp-1	cpt-1	pgc-1	atgl
27 °C	0.35%	1.00 ± 0.17	1.00 ± 0.10 ^CD^	1.00 ± 0.42	1.00 ± 0.30	1.00 ± 0.29
	0.55%	0.88 ± 0.09	0.85 ± 0.05 ^ABC^	1.77 ± 0.37	1.54 ± 0.47	1.14 ± 0.25
	0.71%	0.66 ± 0.14	0.61 ± 0.03 ^A^	2.52 ± 0.32	1.82 ± 0.53	2.07 ± 0.47
	0.82%	0.85 ± 0.07	0.75 ± 0.02 ^ABC^	2.03 ± 0.29	1.74 ± 0.24	1.87 ± 0.73
	0.92%	0.92 ± 0.13	0.81 ± 0.01 ^ABC^	1.78 ± 0.10	1.46 ± 0.47	1.79 ± 0.57
33 °C	0.35%	1.41 ± 0.14	1.41 ± 0.06 ^E^	0.95 ± 0.09	1.09 ± 0.12	0.93 ± 0.08
	0.55%	1.09 ± 0.10	1.16 ± 0.06 ^DE^	1.72 ± 0.07	1.56 ± 0.42	0.96 ± 0.22
	0.71%	0.78 ± 0.06	0.72 ± 0.14 ^AB^	2.24 ± 0.37	1.66 ± 0.52	1.59 ± 0.18
	0.82%	0.93 ± 0.06	0.85 ± 0.15 ^ABC^	1.99 ± 0.18	1.44 ± 0.27	1.28 ± 0.12
	0.92%	1.03 ± 0.14	0.90 ± 0.09 ^BC^	1.61 ± 0.22	1.35 ± 0.31	1.07 ± 0.13
Temperature						
27 °C		0.86 ± 0.15	0.81 ± 0.13	1.82 ± 0.58	1.51 ± 0.46	1.57 ± 0.60 *
33 °C		1.05 ± 0.23 *	1.01 ± 0.26 *	1.70 ± 0.48	1.42 ± 0.36	1.17 ± 0.28
Phosphorus						
0.35%		1.20 ± 0.26 ^c^	1.20 ± 0.23 ^d^	0.97 ± 0.27 ^a^	1.04 ± 0.21	0.96 ± 0.19 ^a^
0.55%		0.98 ± 0.14 ^b^	1.00 ± 0.17 ^c^	1.75 ± 0.24 ^b^	1.55 ± 0.40	1.05 ± 0.23 ^a^
0.71%		0.72 ± 0.11 ^a^	0.67 ± 0.11 ^a^	2.38 ± 0.34 ^c^	1.74 ± 0.48	1.83 ± 0.41 ^b^
0.82%		0.89 ± 0.07 ^ab^	0.81 ± 0.11 ^ab^	2.01 ± 0.22 ^bc^	1.59 ± 0.28	1.58 ± 0.56 ^ab^
0.92%		0.98 ± 0.13 ^b^	0.85 ± 0.08 ^bc^	1.70 ± 0.18 ^b^	1.40 ± 0.36	1.43 ± 0.54 ^ab^
*p* value						
Temperature		<0.001	<0.001	0.261	0.513	0.007
Phosphorus		<0.001	<0.001	<0.001	0.058	0.003
Interaction		0.136	0.076	0.925	0.913	0.514

Significant differences (*p* < 0.05) are highlighted in a column with different symbols (capital letters, simple effects analysis; “*”, water temperature; lowercase letters, phosphorus levels).

**Table 5:** Serum antioxidant parameters of spotted seabass fed diets with different available phosphorus levels and raised at 27 °C and 33 °C.

		CAT ^a^	SOD ^b^	MDA ^c^	GSH-PX ^d^	T-AOC ^e^
27 °C	0.35%	3.58 ± 025	14.93 ± 1.39	23.16 ± 0.87	271.04 ± 87.35	0.84 ± 0.04
	0.55%	4.20 ± 0.41	21.38 ± 1.17	18.06 ± 0.37	435.93 ± 113.12	0.93 ± 0.02
	0.71%	4.61 ± 0.20	25.73 ± 1.98	12.59 ± 0.98	525.79 ± 14.09	1.03 ± 0.02
	0.82%	4.40 ± 0.79	25.87 ± 2.73	13.88 ± 0.97	524.80 ± 49.35	0.97 ± 0.03
	0.92%	4.40 ± 0.66	21.71 ± 2.04	16.08 ± 0.32	457.86 ± 15.83	0.93 ± 0.01
33 °C	0.35%	5.10 ± 0.54	18.15 ± 1.48	26.54 ± 1.42	363.73 ± 142.44	0.97 ± 0.02
	0.55%	5.29 ± 0.54	24.21 ± 1.57	21.07 ± 1.00	577.06 ± 123.58	1.00 ± 0.03
	0.71%	5.98 ± 0.73	30.59 ± 2.19	16.84 ± 1.77	672.30 ± 27.26	1.11 ± 0.03
	0.82%	5.63 ± 1.02	27.33 ± 111	17.98 ± 0.22	639.66 ± 39.76	1.10 ± 0.03
	0.92%	5.58 ± 1.91	20.44 ± 0.61	18.81 ± 1.82	598.08 ± 23.79	0.99 ± 0.02
Temperature						
27 °C		4.24 ± 0.57	21.92 ± 4.44	16.75 ± 3.89	443.09 ± 112.37	0.94 ± 0.07
33 °C		5.52 ± 0.97 *	24.14 ± 4.83 *	20.25 ± 3.74 *	570.16 ± 134.43 *	1.02 ± 0.06 *
Phosphorus						
0.35%		4.34 ± 0.92	16.54 ± 2.18 ^a^	24.85 ± 2.13 ^d^	317.38 ± 117.23 ^a^	0.91 ± 0.07 ^a^
0.55%		4.74 ± 0.73	22.79 ± 1.98 ^b^	19.56 ± 1.78 ^c^	506.49 ± 131.16 ^b^	0.96 ± 0.04 ^bc^
0.71%		5.30 ± 0.89	28.16 ± 3.25 ^c^	14.71 ± 2.66 ^a^	599.05 ± 82.56 ^b^	1.07 ± 0.04 ^d^
0.82%		5.01 ± 1.06	26.59 ± 2.03 ^c^	15.93 ± 2.34 ^ab^	582.23 ± 74.59 ^b^	1.00 ± 0.05 ^c^
0.92%		4.99 ± 1.44	21.07 ± 1.52 ^b^	17.44 ± 1.90 ^b^	527.97 ± 78.90 ^b^	0.96 ± 0.03 ^b^
*p* value						
Temperature		0.001	0.002	<0.001	<0.001	<0.001
Phosphorus		0.393	<0.001	<0.001	<0.001	<0.001
Interaction		0.992	0.067	0.709	0.971	0.225

Significant differences (*p* < 0.05) are highlighted in a column with different symbols (“*”, water temperature; lowercase letters, phosphorus levels). ^a^ Catalase (U/mL). ^b^ Superoxide dismutase (U/mL). ^c^ Malondialdehyde (nmol/mL). ^d^ Glutathione peroxidase (U/mL). ^e^ Total antioxidant capacity (mmol/L).

**Table 6:** Intestinal microbiota composition of spotted seabass fed diets with different available phosphorus levels and raised at 27 °C and 33 °C.

		Phylum		Genus		
		Proteobacteria	Firmicutes	*Plesiomonas*	*Bacillus*	*Lactococcus*
27 °C	0.35%	91.68 ± 1.36	7.93 ± 1.50	78.05 ± 5.10	5.46 ± 1.48 ^A^	2.45 ± 0.59 ^A^
	0.71%	65.40 ± 5.10	32.83 ± 9.71	47.87 ± 3.10	7.32 ± 0.90 ^A^	26.02 ± 6.90 ^B^
	0.92%	71.11 ± 9.20	28.67 ± 6.16	42.61 ± 5.35	7.60 ± 2.40 ^A^	18.00 ± 3.10 ^B^
33 °C	0.35%	84.75 ± 1.18	12.01 ± 1.39	76.53 ± 5.35	6.40 ± 1.10 ^A^	2.40 ± 0.19 ^A^
	0.71%	56.78 ± 10.49	43.01 ± 10.52	42.58 ± 6.62	20.51 ± 3.80 ^B^	4.69 ± 0.59 ^A^
	0.92%	72.32 ± 11.22	27.04 ± 10.79	51.96 ± 6.75	27.64 ± 5.49 ^B^	3.50 ± 0.61 ^A^
Temperature						
27 °C		70.06 ± 13.09	23.14 ± 12.92	56.17 ± 17.04	6.79 ± 1.79	15.49 ± 11.04 *
33 °C		72.28 ± 15.46	27.34 ± 15.41	57.02 ± 16.12	18.18 ± 9.95 *	3.52 ± 1.08
Phosphorus						
0.35%		89.71 ± 2.43 ^b^	9.97 ± 2.58 ^a^	77.29 ± 4.75 ^b^	5.93 ± 1.27 ^a^	2.43 ± 0.40 ^a^
0.71%		61.09 ± 8.76 ^a^	37.91 ± 10.63 ^b^	45.22 ± 5.45 ^a^	13.92 ± 7.63 ^b^	15.36 ± 12.47 ^b^
0.92%		71.72 ± 9.20 ^a^	27.84 ± 7.91 ^b^	47.29 ± 7.48 ^a^	17.62 ± 11.61 ^b^	10.75 ± 8.18 ^b^
*p* value						
Temperature		0.314	0.275	0.750	<0.001	<0.001
Phosphorus		<0.001	<0.001	<0.001	<0.001	<0.001
Interaction		0.554	0.445	0.097	<0.001	0.029

Significant differences (*p* < 0.05) are highlighted in a column with different symbols (capital letters, simple effects analysis; “*”, water temperature; lowercase letters, phosphorus levels).

## Data Availability

The data presented in this study are available on request from the corresponding author.

## Supplementary Materials

The following supporting information can be downloaded at: https://www.mdpi.com/article/10.3390/antiox12122128/s1, Table S1. Formulation and proximate composition of experimental diets. Table S2. Sequences of primers used for RT-qPCR.
