# Supplementary material for: Physiological Response of Spotted Seabass (Lateolabrax maculatus) to Different Dietary Available Phosphorus Levels and Water Temperature: Changes in Growth, Lipid Metabolism, Antioxidant Status and Intestinal Microbiota
Source: Antioxidants (Basel). 2023 Dec 16;12(12):2128. doi: 10.3390/antiox12122128 (PMC10740591; doi:10.3390/antiox12122128)
Supplement: Supplementary file 1 [file antioxidants-12-02128-s001.zip › antioxidants-2760923-supplementary.pdf]

Supplementary material

Table S1. Formulation and proximate composition of experimental diets.

Ingredients (%, dry basis)	0.35%P	0.55%P	0.71%P	0.82%P	0.92%P
Deboned fishmeal	15.00	15.00	15.00	15.00	15.00
Casein	22.00	22.00	22.00	22.00	22.00
Wheat gluten	8.00	8.00	8.00	8.00	8.00
Corn dextrin	35.00	35.00	35.00	35.00	35.00
Squid paste	3.00	3.00	3.00	3.00	3.00
Fish oil	7.00	7.00	7.00	7.00	7.00
Soybean oil	3.00	3.00	3.00	3.00	3.00
Lecithin	2.00	2.00	2.00	2.00	2.00
Mineral premix[a]	0.50	0.50	0.50	0.50	0.50
Vitamin premix[b]	0.40	0.40	0.40	0.40	0.40
Vitamin C	0.10	0.10	0.10	0.10	0.10
Choline chloride	0.50	0.50	0.50	0.50	0.50
Taurine	0.10	0.10	0.10	0.10	0.10
Y_2O_3	0.10	0.10	0.10	0.10	0.10
Microcrystalline cellulose	3.52	3.22	2.91	2.71	2.51
NaH_2PO_4	0.00	0.49	0.98	1.31	1.64
K_2HPO_4	0.00	0.35	0.69	0.93	1.16
KCl	1.00	0.70	0.41	0.20	0.00
NaCl	0.78	0.54	0.30	0.15	0.00
**Proximate composition**					
Crude protein	43.74	43.21	43.92	43.16	43.27
Crude lipid	12.45	12.37	12.38	12.63	12.46
Phosphorus (%)	0.36	0.56	0.72	0.83	0.94
Available phosphorus (%)	0.35	0.55	0.71	0.82	0.92

[a]Vitamin premix (mg/kg diet): VB_1, 10; riboflavin, 8; pyridoxine HCl, 10; VB_{12}, 0.2; VK_3, 10; inositol, 100; pantothenic acid, 20; niacin acid, 50; folic acid, 2; biotin, 2; VA (500,000 IU), 400; VD_3, 5; VE (500,000 IU), 100; ethoxyquin, 150; wheat middling, 3132.8.

[b]Mineral premix (mg/kg diet): $MgSO_4{\cdot}H_2O$, 2000; $MnSO_4{\cdot}H_2O$, 40; $ZnSO_4{\cdot}H_2O$, 150; $CuSO_4{\cdot}5H_2O$, 15; $FeSO_4.H_2O$, 230; Na_2SeO_3 (1%), 50; $CoSO_4$ (5%), 20; KI (1%), 100; Zeolite, 2395.

Table S2. Sequences of primers used for RT-qPCR.

Target gene	Primer sequence (5'-3')	Amplicon length (bp)	Amplification efficiency
β-actin	F: CAACTGGGATGACATGGAGAAG<br>R: TTGGCTTTGGGGTTCAGG	159	102.65%
napiiia	F: GAAGAGGATAAGTGGGGAGACG<br>R: AAATGGAGGCTGAAGCGAAG	138	99.94%
napiiib	F: GGTGTTAGTCACAGTGGGGG<br>R: GGTGACAGACGTTCCGATGT	146	98.57%
pit1	F: CTGCTTCCTCCCCATCAACA<br>R: GGATAACCCACACACCAAACCA	141	99.38%
pit2	F: ATCACGAGGAGAAGGATAAGCC<br>R: CATTACACCGCCCTGGTCATA	104	99.79%
pgc-1	F: GTTCCTCCGAACTCCCAGTG<br>R: CAGACGCTCACTCCTTGTGT	129	96.20%
atgl	F: CTTCCTCTCCGCAACAAGTC<br>R: TGGTGCTGTCTGGAGTGTTC	151	98.79%
cpt-1	F: CCTCAATGATACATCGGAACCC<br>R: CTGCGGCTCATCATCTAACG	124	99.42%
fas	F: AAACTGAAGCCCTGTGTGCC<br>R: CACCCTGCCTATTACATTGCTC	169	96.53%
srebp-1c	F: CCTCACTCTGCAGCCAATCA<br>R: CGTAGTCCCACCCTCAAACC	130	99.12%

napiiia, sodium-phosphate cotransporter iia; *napiiib,* sodium-phosphate cotransporter iib; *pit1,* sodium-phosphate cotransporter iic 1; *pit2,* sodium-phosphate cotransporter iic 2; *pgc-1,* peroxisome proliferator-activated receptor-gamma coactivator 1; *atgl,* adipose triglyceride lipase; *cpt-1,* carnitine palmitoyltransferase 1; *fas,* fatty acid synthetase; *srebp-1c,* sterol-regulatory element binding proteins 1c.
